# Study Design and Protocol of a Randomized Controlled Trial of the Efficacy of a Smartphone-Based Therapy of Migraine (SMARTGEM)

**DOI:** 10.3389/fneur.2022.912288

**Published:** 2022-06-16

**Authors:** Ana Sofia Oliveira Gonçalves, Inga Laumeier, Maxi Dana Hofacker, Bianca Raffaelli, Philipp Burow, Markus A. Dahlem, Simon Heintz, Tim Patrick Jürgens, Steffen Naegel, Florian Rimmele, Simon Scholler, Tobias Kurth, Uwe Reuter, Lars Neeb

**Affiliations:** ^1^Institute of Public Health, Corporate Member of Freie Universität Berlin, Charité—Universitätsmedizin Berlin, Humboldt Universität zu Berlin, Berlin, Germany; ^2^Department of Neurology, Corporate Member of Freie Universität Berlin, Charité—Universitätsmedizin Berlin, Humboldt Universität zu Berlin, Berlin, Germany; ^3^Kath. Marienkrankenhaus gGmbH, Hamburg, Germany; ^4^Clinician Scientist Program, Berlin Institute of Health (BIH), Berlin, Germany; ^5^Department of Neurology, University Hospital Halle, Martin-Luther-University Halle-Wittenberg, Wittenberg, Germany; ^6^Newsenselab GmbH, Berlin, Germany; ^7^Department of Neurology, University of Rostock, Rostock, Germany; ^8^Universitätsmedizin Greifswald, Greifswald, Germany

**Keywords:** migraine, eHealth, smartphone application (app), integrated care, teleconference

## Abstract

**Background:**

Digitalization and electronic health (eHealth) offer new treatment approaches for patients with migraine. Current smartphone applications (apps) for migraine patients include a wide spectrum of functions ranging from digital headache diaries to app-based headache treatment by, among others, analysis of the possible triggers, behavioral therapy approaches and prophylactic non-drug treatment methods with relaxation therapy or endurance sport. Additional possibilities arise through the use of modern, location-independent communication methods, such as online consultations. However, there is currently insufficient evidence regarding the benefits and/or risks of these electronic tools for patients. To date, only few randomized controlled trials have assessed eHealth applications.

**Methods:**

SMARTGEM is a randomized controlled trial assessing whether the provision of a new digital integrated form of care consisting of the migraine app M-sense in combination with a communication platform (with online consultations and medically moderated patient forum) leads to a reduction in headache frequency in migraine patients, improving quality of life, reducing medical costs and work absenteeism (DRKS-ID: DRKS00016328).

**Discussion:**

SMARTGEM constitutes a new integrated approach for migraine treatment, which aims to offer an effective, location-independent, time-saving and cost-saving treatment. The design of the study is an example of how to gather high quality evidence in eHealth. Results are expected to provide insightful information on the efficacy of the use of electronic health technology in improving the quality of life in patients suffering from migraine and reducing resource consumption.

## Introduction

Migraine is a primary headache disorder affecting ~15% of people worldwide (1). The Global Burden of Disease 2015 study lists migraine as the number one source of disability caused by a neurological disease (2). Migraines can disrupt patients' daily life and diminish their quality of life. Moreover, migraine is the first cause of disability in patients under 50 and often leads to an incapacity to work in these productive years (3).

Nevertheless, patients suffering from migraine face numerous barriers to effective migraine care, including not being able to consult a specialist, failure to receive a correct diagnosis, and not being prescribed a regimen with acute and preventive pharmacologic treatments (4). Multidisciplinary treatment approaches have been shown to be an efficient therapeutic option in headache disorders (5). Patients with migraine benefit from cognitive behavioral interventions (6, 7), but waiting lists for psychotherapy appointments in Germany are long and access is further restricted in rural areas.

Self-management programmes have shown to be effective for long-term conditions (8). In the case of headaches, such programmes can help patients take an active role in the management of their disease, by learning more about the condition and through behavioral changes (9). Several non-pharmaceutical behavioral interventions such as relaxation therapy and endurance sports are important for migraine management but need instruction and patients' motivation (10). Moreover, the recurring nature of this condition often leads to the frequent intake of medication, which itself may further increase headache frequency, known as medication overuse headache (11). Education in non-pharmacological prevention is thus key to prevent chronification and medication overuse (12).

Digital healthcare tools can help, at least partially, to address the aforementioned challenges.

The interest in smartphone applications (“apps”) that are used for diagnostics or treatment of medical conditions has been increasing in various medical fields (13, 14). While there are many smartphone applications for headache disorders, high-quality studies that evaluate their efficacy and possible benefit (or harm) are still lacking. A review on mobile apps for the management of headache disorders by Mosadeghi-Nik et al. found a low level of clinical evidence for (at this time) existing smartphone applications that included a diary function and intervention elements (15). However, authors and reviewers considered smartphone applications to have a high potential for the improvement of care for headache patients (16–18). Further communication tools such as expert chats and patients' forums offer easy access to information and counseling and may also be effective in migraine treatment.

The purpose of SMARTGEM (smartphone assisted migraine therapy) is to assess with a randomized controlled trial if the provision of digital tools in migraine therapy may lead to a reduction of migraine frequency, improve quality of life, reduce medical costs and prevent work absenteeism in patients with migraine. SMARTGEM is a new digital integrated form of care that consists of the medically certified migraine app M-sense in combination with a communication platform (with online consultations and a medically moderated patient forum).

## Methods

### Hypothesis

The implementation of SMARTGEM, consisting of the certified medical app for migraine (M-sense) in combination with a communication platform (with online consultations and medically moderated patient forum), leads to a decrease in monthly migraine days in patients with migraine with at least 5 migraines days per month after 6 month.

### Intervention and Control

The SMARTGEM intervention consists of three elements:

#### M-Sense app

M-sense is a medically certified app that was developed independently of SMARTGEM and prior to the project start by an interdisciplinary team of scientists, psychologists, app developers and economists (Newsenselab GmbH) in cooperation with physicians.

Patients in the intervention group had access to all the functions of the M-sense app (called M-sense active). The app included a headache diary, where participants can fill in their headache attacks' characteristics. This comprises the start and endpoint of the headache attack, maximal pain intensity, whether the headache is unilateral, whether it is a throbbing headache, if an aura is present, as well as information on concomitant symptoms—aggravation due to physical activity, aura, vomiting, nausea, phonophobia, and photophobia. Furthermore, for each headache attack, patients may document the intake of acute medication, including the name and dose of the drug and time of intake. The smartphone can also add information on weather data (temperature, air pressure, etc.) to the diary.

The app classified each headache attack as migraine, tension-type headache (TTH), or non-migraine or non-TTH, using a standardized, validated algorithm (19) based on the International Classification of Headache Disorders (ICHD-3) criteria (20). A migraine day was defined as a calendar day on which the patient suffered from a qualified migraine attack. A headache day was defined as any calendar day on which the patient experienced a headache attack (including migraine).

To identify if an attack should be considered as a migraine or a TTH, all relevant ICHD-3 criteria were applied by the algorithm (19). A qualified migraine attack needed to fulfill the following ICHD-3 criteria (20) for being classified as migraine: a headache with or without aura lasting at least 4h with both features A (at least 2 of the following: unilateral location, pulsatile quality, moderate or severe pain intensity, and aggravation caused by physical activity or avoidance of physical activity) and B (during headache, at least 1 of the following: nausea and/or vomiting and/or photophobia and phonophobia). Headache attacks accompanied by the intake of migraine-specific medication (such as triptans) to treat a headache or accompanied by aura were considered as migraine, regardless of the duration and pain characteristics or concomitant symptoms.

In addition to the attacks' characteristics as well as their symptoms, patients could also document their sleep patterns, stress levels, skipped meals, coffee and/or alcohol consumption. The app version available to patients in the intervention group analyzed these factors with the aim of defining a possible connection with the patient's headache patterns, thus identifying individual triggers.

For patients in the intervention group, the app also offered preventive modules that enable the patient to take an active role in their migraine management (21). The therapy module offered different relaxation methods — such as progressive muscle relaxation according to Jacobson or autogenic training — as well as created individual training programmes for endurance sports. Another module offered animated physio-therapeutic exercises. Patients could access these according to a personalized plan, but in the case of acute headaches, they could also get help in an acute module via guided imagination exercises and physiotherapy exercises. In an educational module, patients could deepen their knowledge about migraine via a “chatbot” and learn basic behavioral therapy approaches in migraine therapy.

#### Patient's Forum and Experts' Chat

Patients had access to a secured web based communication platform with an individual login. Via this platform, they could consult the physicians of the participating headache centers using an expert chat. Each center provided several time slots per week for synchronous communication. Patients could also ask their questions outside these time slots and were provided with an answer during the next 24 h (asynchronous communication). A patients' forum moderated by the physicians of the participating headache centers was also provided.

However, SMARTGEM did not allow continuous medical support 24/7 in the current concept. In case of an emergency like refractory migraine attack, persisting aura or unusual headache symptoms all patients were instructed to introduce themselves immediately at the emergency department of the participating centers (which provide a neurologist on call 24/7) or another hospital nearby.

#### Telemedicine

Physicians of the participating headache centers had access to the digital headache diary of their patients. This enabled the physicians to online monitor the course of the migraine during treatment and could be used for online consultations.

Furthermore, resident doctors providing the ongoing treatment of patients (neurologists, pain specialists or general practitioners) could consult with headache experts of the headache centers and discuss treatment based on the digital headache diary.

### Study Overview

**Figure 1:**
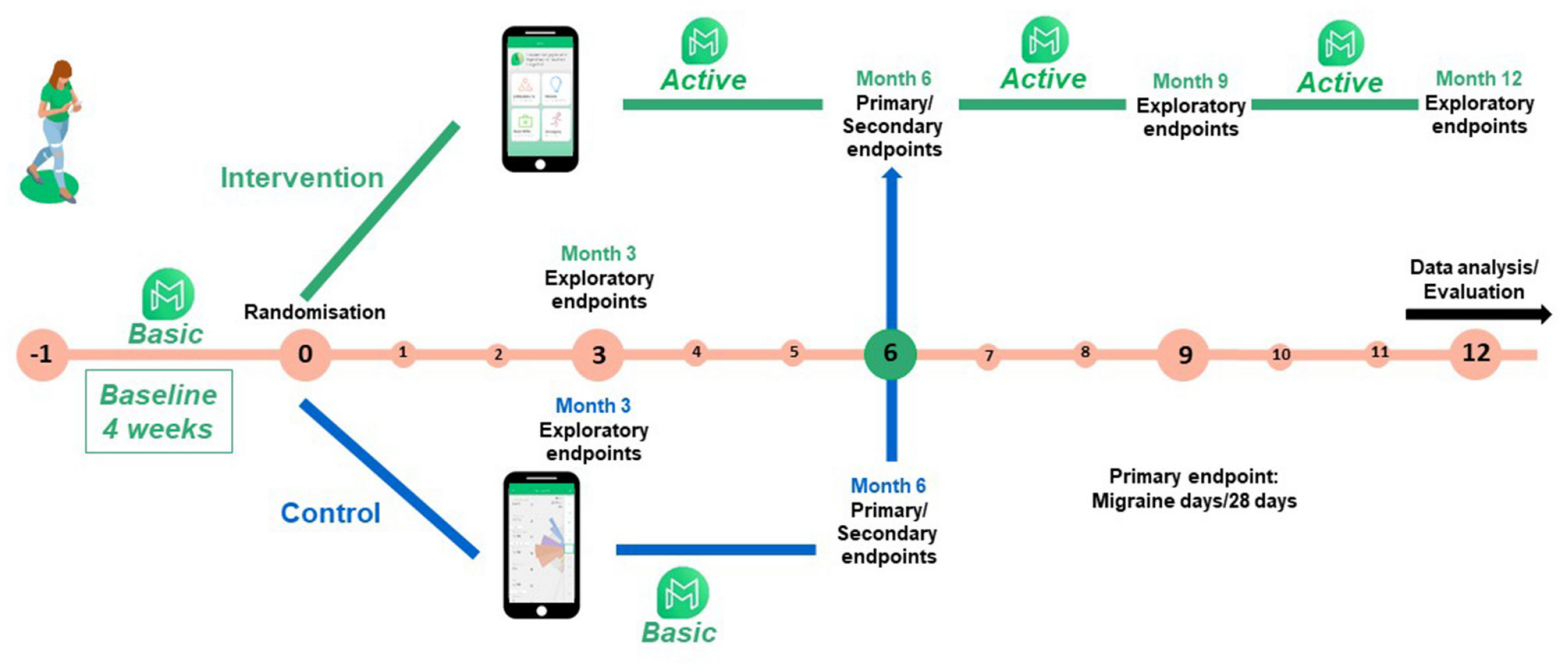
SMARTGEM's study flow.

SMARTGEM is a multi-center, two-armed, open-label, randomized controlled trial comparing standard of care plus the use of a certified medical app for migraine (M-sense) in combination with a communication platform (with online consultations and medically moderated patient forum) vs. standard of care plus a basic version of M-sense app with a headache documentation function only. The study design was developed following the guidelines of the International Headache Society for controlled trials of prophylactic treatment in migraine (22). Figure 1 outlines the study flow and the timeline of SMARTGEM.

### Recruitment and Study Participants

Adult patients (≥18 years old) previously diagnosed with migraine with less than three prior visits to the outpatient headache centers were recruited from the three participating sites (Charité — Universitätsmedizin Berlin, Universitätsmedizin Rostock and Universitätsklinikum Halle). Additionally, any adult patient who was scheduled for a first visit in the headache outpatient centers and was interested in participating could go through a telephone pre-screening. Successfully pre-screened patients got access to the basic functions of the M-sense app and were asked to fill in the app diary on a daily basis for 28 days previously to their first appointment with a headache specialist and clinical investigator (baseline). To be eligible for trial inclusion, patients had to have a migraine according to the ICHD-3 criteria (20) with at least five migraine days during the 28 day pre-baseline phase and a headache diary compliance of at least 80%. Further inclusion and exclusion criteria are described in the Supplementary Table A1. Only patients that gave their written informed consent were included in the study

After the pre-baseline phase, all patients that still met the inclusion criteria were randomly assigned to the intervention or the control group. Patients in the intervention group received standard care and all functions of SMARTGEM. Patients in the control group received standard care together with a basic version of M-sense app that serves only as an electronic headache diary.

SMARTGEM also included patients who were planned for participation in an existing multimodal treatment programme (KopfschmerzSPEZIAL) at the Charité — Universitätsmedizin Berlin. Patients in this programme were evaluated with regard to neurologic, psychological and physiotherapeutic aspects and a treatment plan was customized according to their needs. Furthermore, severely affected patients underwent a multi-modal, outpatient therapy programme, including, among others, group sessions and one-to-one discussions, guidance on endurance sports and individual physiotherapy training.

Potential participants were recruited over a 23 month period (January 2019 to December 2020). Participants were followed for 12 months.

### Study Objectives and Outcomes

A list of the primary and the secondary outcomes is presented in Table 1. All primary and secondary outcomes will be assessed at month 6 in comparison to baseline and between groups.

**Table 1:** Study outcomes and data collection method.

**Outcomes**	**Data source**	**Timepoint**
*Primary Outcome* Migraine days per 28 days	M-sense electronic diary	Baseline vs. month 6
*Secondary outcomes*		
Responder rate (patients with ≥50% reduction from baseline in migraine days/month)	M-sense electronic diary	First six months in the study
Headache days per 28 days	M-sense electronic diary	Baseline vs. month 6
Days of acute medication intake	M-sense electronic diary	Baseline vs. month 6
HIT-6 score	Study databank	Baseline vs. month 6
EQ-5D-5L score	Study databank	Baseline vs. month 6
PROMIS 29 scores	Study databank	Baseline vs. month 6
Overall health care costs	Claims' data	Six months prior study initiation versus six months after study initiation
Days with inability to work	Claims' data	Six months prior study initiation versus six months after study initiation

*HIT-6, Headache Impact-Test 6; PROMIS 29, Patient-Reported Outcome-measurement Information System*.

The primary outcome is the reduction of migraine days per 28 days from baseline, as measured 6 months after inclusion in the study. Using data collected by M-sense, we will analyze the secondary endpoints regarding the number of patients with at least 50% reduction in migraine days per 28 days, the reduction of headache days per 28 days, as well as days of acute medication intake. Patient-related outcome measures will be assessed with validated surveys. In order to evaluate the impact of SMARTGEM on patients' quality of life we collect data with the Headache Impact-Test 6 (HIT-6) (23), Patient-Reported Outcome-measurement Information System 29 (PROMIS 29) (24) and German EQ-5D−5L (25). Furthermore, we will assess depression, anxiety and tension/stress of the patients with the depression anxiety stress scale (DASS) (26) and patients adherence with the Medication Adherence Report Scale (MARS-D) (27).

The aforementioned endpoints will also be analyzed at the follow-up periods (month 3, 6, 9 and 12), but in an explorative fashion only, i.e., without formally testing hypotheses by statistical tests. Additional exploratory endpoints are listed in the Supplementary Table A2.

Claims' data from the participating statutory health insurance will be collected to analyze participants' healthcare resources' consumption and work absenteeism.

Exploratory subgroup analyses for the primary and secondary outcomes will be carried out. This is intended to provide insights about which groups of patients particularly benefit from SMARTGEM with regard to their chronification stage:

- Episodic migraine
  ○ Medium-frequency episodic migraine (between 5 and 7 migraine days per month and less than 15 headache days per month)
  ○ High-frequency episodic migraine (at least 8 migraine days per month and less than 15 headache days per month)
  - Chronic migraine
    ○ Chronic migraine without medication overuse
    ○ Chronic migraine with medication overuse

Chronic migraine was defined according to the ICHD-3 criteria (20). Medication overuse was defined as a) use of conventional analgesics (non-opioid analgesics such as non-steroidal anti-inflammatory drugs (NSAIDs) or paracetamol on at least 15 days/months or b) triptans on at least 10 days/months or c) combination therapy (any combination of triptans, ergotamines and analgesics) on at least 10 days per month. We used the definition from Tepper et al. (28). It should be noted that this definition should not be confused with the diagnosis of medication overuse headache. The subgroups were developed using patients' baseline data and the diagnosis of the physician according to the first study visit. Patients with chronic migraine had to have at least 15 headache days per month, of which at least 8 were migraine for >3 m before screening and assessed by the M-sense headache diary during the baseline period.

Physicians record data on patients' pharmacological and non-pharmacological therapies. Further study-specific questionnaires are used to obtain additional information as regards the satisfaction with the programme from the patients' and physicians' perspective.

We used the REDCap software to collect and record data (29).

### Randomization

Participants were randomized to the intervention group or the control group using a randomized block design with a block length of 50 and an allocation ratio of 1:1. The randomization list was uploaded to the project's electronic data capture system, REDCap (29), where the randomization was conducted.

Before randomization, patients were informed about what the intervention entailed, and it was highlighted that control group patients could receive the intervention after 6 months in the study. Since the control group had no access to the extended app functions and no access to the communication platform, the test subjects could not be blinded to the intervention.

### Sample Size Calculation

The study aimed to recruit 576 patients. The sample size calculation was based on the primary outcome, migraine days per 28 days after 6 months in comparison between the intervention and the control group.

A mixed-effects linear regression was fitted, with the dependent variable being the number of migraine days at baseline and the independent variables being treatment group and migraine days at 6 months. In addition, we included a random coefficient for treatment exposure and a random intercept for regions. Power was calculated as the percentage where the *p*-value was ≤0.05 in the 150 simulation runs. With these assumptions, we achieved a power of 80% with 500 patients. Assuming a 13% dropout rate, 576 patients need to be enrolled. The software R version 3.6.3. was employed to perform this calculation (30).

### Statistical Analysis

The data will be evaluated according to the intention-to-treat principle (ITT). A Per Protocol (PP) analysis will also be conducted.

Patients' baseline characteristics will be described by assigned group. The primary outcome will be assessed with a mixed-effects linear regression, with the dependent variable being the number of migraine days at the start of the study and the independent variables being the treatment group and the migraine days after 6 months. We also included a random intercept for treatment and a random coefficient for the region. If normally distributed, secondary outcomes will be compared between groups using two-sided *t*-tests. If data are not normally distributed, group differences will be analyzed using a Brunner-Munzel test (31). If an adjustment of baseline covariates is necessary, linear regressions with appropriate adjustments will be carried out. Chi-Square tests or the Brunner-Munzel tests will compare categorical endpoints. In order to assess the endpoints over time, multivariate analyses will be conducted using mixed-effects models. These models will also serve to estimate possible effects of covariables (e.g., socio-demographic characteristics) on the endpoints.

Effects will be analyzed within subgroup categories and across subgroups. In the first case, this means that in each subgroup, the effect of the intervention is analyzed after 6 m with regard to a specific endpoint (e.g., number of days with migraines). The following effects across subgroups will be compared: episodic migraine vs. chronic migraine; medium-frequency episodic migraine vs. high-frequency episodic migraine; chronic migraine with drug overuse vs. chronic migraine without drug overuse. In order to test for a statistically significant heterogeneity of the effect of the intervention (compared to the control group) on the selected endpoints, the *p*-value of the interaction term in the model is used. We differentiate between frequency interactions (episodic, medium-frequency, high-frequency, chronic) and drug-induced. Since these analyses are explorative, they will not be adjusted for multiple testing.

For the primary outcome, a two-tailed *p*-value with a significance level ≤0.05 will be considered significant. For the secondary endpoints, we will account for multiple testing with a Bonferroni adjustment. Data analysis will be performed using R 4.0.2 (30).

## Discussion

The presented trial SMARTGEM aims to assess the efficacy of a digital intervention in migraine therapy in a randomized controlled trial.

Digital healthcare tools have the potential to help, at least partially, to address several challenges that migraine sufferers currently face, such as the difficulties to assess specialist consultations, insufficient knowledge about the disease and the unfamiliarity with non-pharmaceutical behavioral interventions. However, high quality studies that assess the efficacy of these digital interventions are lacking. Even worse, many apps make scientific claims on the app stores, but only a small minority of these apps can offer clinical evidence with mostly low-quality. There is a striking discrepancy between the availability of many commercial migraine apps and the few studies that may serve to create high level evidence of the efficacy of these apps (16–18).

In the last few years, few studies have started to provide evidence for the use of smartphone applications in migraine therapy. Two randomized controlled trials could show that an app-based heart rate variability biofeedback and a smartphone delivered progressive muscle relaxation are feasible and acceptable for people with migraine. However, a clinical significant effect could not be shown in both studies. These might be due to the low number of participants, but also to the use of the app by the participants. High frequency users of the app reported more headache free days than low frequency users (32). An observational study from 2019 carried out in Germany analyzed the use of an app (Migräne-App) as part of the care for migraine and headache patients (33). Users reported that the migraine app helped them to follow their treatment plan, they had a highly significant reduction of headache days, as well as a highly significant reduction of acute medication intake. In contrast to SMARTGEM, in this study, participants self-reported their app usage, the course of their headache disorders and previous treatments in an online survey, while in our study this information was analyzed directly from recorded data in the app. Specifically in terms of app usage, M-sense automatically calculates how frequently participants actually use the app. Thus, there is less potential for biased answers by the participants. Another German observational study assessed the evolution of headache using the basic version of the M-sense app (34). It found that headache and migraine frequency decreased over time for regular users of the electronic headache diary. While this study was important to analyze headache trends over time in users of an electronic headache diary, it did not compare in a controlled manner the use of the basic version of the app with a new digital integrated form of care like SMARTGEM.

Although relating to another pain disorder, the Rise-uP cluster-randomized trial assessed the treatment of back pain with a new digital integrated form of care which also included an app (Kaia app) and teleconsultations (33). The authors found that the intervention group had a significantly stronger pain reduction compared to the control group after 3 months.

Since new technologies in healthcare are inevitable, it is necessary to critically analyze their benefits but also their potential risks. While current pain management apps and most of the available headache apps include evidence-based headache management behavior change techniques with focus on self-monitoring of stress management, high quality studies such as randomized controlled trials are necessary to determine their efficacy.

The presented trial SMARTGEM will analyze digital health tools that have the potential to help manage migraine prevention and treatment in a randomized controlled environment. The app M-sense aims to motivate patients to take an active part in their treatment and recovery in terms of non-pharmacological approaches, self-management and behavior change techniques. Included components of self-management are self-monitoring, patient education, self-care skills instruction and goal-setting. The online platform with the patient's forum complements a social support function (35). The expert chat allows the patient to seek the help of a headache expert without long waiting times and the need to come into office. This may facilitate access to physicians and help to solve time critical problems such as management of side effects of a new acute oral preventive medication. To our knowledge there are no studies evaluating the effect of consultations via chat in migraine patients. A Swedish study in a primary care setting found that patients enjoyed the more anonymous character of these consultations, although others preferred face-to-face meetings. An important advantage of this tool is that it allows patients to refer back to what had been said by them and the physician (36). There is preliminary evidence in favor of self-management in headache medicine (37) and for other pain conditions such as low back pain (38). Self-care skills instruction and goal-setting in SMARTGEM include relaxation techniques such as progressive muscle relaxation and endurance sports. There is good evidence for the use of relaxation techniques in migraine prevention and the benefit of aerobic endurance sport was also provided by some studies (10, 39). Both measures are commonly recommended as an important part of migraine preventive therapy. However, adherence to these tools is difficult to monitor and can undermine their efficacy. Mobile based methods may help to measure adherence and to engage individual patient adherence. The app M-sense might motivate the patients to regularly perform these activities by reminders, goal-settings and a reward system and therefore help to reduce migraine frequency.

During the COVID-19 pandemic in-person outpatient health care visits plummeted (40). Thus, measures that allowed access to treatment and management of illnesses were essential. Due to the location-independent nature of SMARTGEM, patients could still benefit from the study. Those in the intervention group could manage their condition with behavioral modules offered in M-sense (relaxation exercises and endurance sports) even during the COVID-19 pandemic. Another study conducted in Italy during the pandemic showed promising results regarding the use of a behavioral approach offered in a smartphone app for the management of chronic migraine with medication overuse (41). Intervention-group patients in SMARTGEM could also continue to use the communication platform (with online consultations and a medically moderated patient forum) safely from their homes. In terms of the study flow, follow-up visits at month six were on site, while during other time points were by telephone. During the COVID-19 lockdown, we offered patients the possibility to do their 6-months visit per telephone, which was appreciated by some patients.

One strength of the study is that its design and its endpoints followed the recommendations of the guidelines for controlled trials in migraine (22). Although these guidelines were developed for the design of drug trials, we believe that the same strict criteria should be applied to digital or other non-pharmacological interventions whenever possible. Limitations of this study are possible bias resulting from the selection of particularly motivated patients and patients with an affinity for smartphone usage. Given answers may in part result from social expectations. One major limitation is that the type of digital intervention does not allow to blind patients to study groups which could increase bias. Claims' data from statutory health insurances in Germany are subject to delays of about 9 month, inherent in their updating since, especially outpatient data, are not promptly communicated. Hence, patients claims' data will only be evaluated within a 6 months period in order to guarantee their analysis within the project duration. A further limitation is that the regular use of a digital headache diary alone might lead to a decrease of headache frequency in migraine patients. This might be explained by a raise of awareness and identification of possible patterns and aggravating factors. This might impede the detection of differences between the control group and the intervention group.

## Conclusion

SMARTGEM constitutes a new digital approach for migraine treatment, which aims to offer an effective, location-independent, time-saving and cost-saving treatment. Results are expected to provide insightful information on the efficacy of the use of electronic health technology to improve the quality of life of patients suffering from migraine and to reduce consumption of healthcare resources and loss of work productivity.

### Trial Status

Recruitment ended in December 2020. First results are expected in the third quarter of 2022.

### Ethics, Informed Consent and Data Protection

The local ethics review board at the Charité — Universitätsmedizin Berlin approved this study (approval number: EA4/110/18). The study will be conducted according to standard guidelines for clinical trials (Declaration of Helsinki, guidelines for good clinical practice). Oral and written informed consent is obtained from every participant. Data protection regulations obey the current German law on data protection.

The study was registered in the German Register of Clinical Studies on 17/12/2018 under the number “DRKS00016328”.

## Ethics Statement

The studies involving human participants were reviewed and approved by Charité—Universitätsmedizin Berlin: EA4/110/18 Universitätsklinikum Halle (Saale): 2018–157 Universitätsmedizin Rostock: A 2018-0191. The patients/participants provided their written informed consent to participate in this study.

## Author Contributions

LN and UR developed the overall concept of the study. MD was involved in the app-specific aspects. LN is the principal investigator. TK is the principal investigator of the evaluation and developing the evaluation for the study. AO, MH, and LN drafted the manuscript. AO and TK prepared the statistical analysis plan. All authors reviewed the draft and provided feedback and approved the final manuscript.

## Funding

SMARTGEM is financially supported by the German Innovation Committee for the promotion of new forms of care. Funded projects shall provide enough evidence to serve as a basis for structural changes in the legal framework and to be permanently integrated into regular care (under paragraph 2, section 92a of Book V of the German Social Security Code) (Grant number: 01NVF17038).

## Conflict of Interest

BR reports research grants from Novartis, and personal fees from Allergan, Hormosan, Lilly, Novartis, and Teva. MD is a Co-founder, managing director, and shareholder of the Newsenselab GmbH (App M-sense). SH received speaking honoraria by TEVA. TJ contributed to advisory boards of Allergan, Hormosan, Lilly, Lundbeck, Novartis, and Teva and received speaking fees from Allergan, Grünenthal, Hormosan, Lilly, Lundbeck, Novartis, Sanofi, and Teva and research funding from Novartis. SN contributed to advisory boards of Hormosan, Lundbeck, Novartis, and TEVA, and received speaking fees from Allergan, Hormosan, Lilly, Novartis, and TEVA. He received research grants from Novartis. FR contributed to advisory boards of AllerganNovartis, TEVA, and received speaking fees from Allergan, Ipsen, Lilly, Novartis, and TEVA. SS is employed at Newsenselab GmbH (App M-sense). TK reported to have received research grants from the German Joint Committee and the German Ministry of Health. He further has received personal compensation from Eli Lilly & Company, Teva, TotalEnergies S.E., the BMJ, and Frontiers. UR received honoraria for consulting and lectures from Amgen, Allergan, Abbvie, Eli Lilly, Lundbeck, Novartis, electroCore, Medscape, StreaMedUp, and Teva, and research funding from the German Federal Ministry of Education and Research and Novartis. LN contributed to advisory boards of Hormosan, Lilly, Novartis, and TEVA, and received speaking fees from Allergan, Hormosan, Lilly, Novartis, and TEVA. The remaining authors declare that the research was conducted in the absence of any commercial or financial relationships that could be construed as a potential conflict of interest.

## Publisher's Note

All claims expressed in this article are solely those of the authors and do not necessarily represent those of their affiliated organizations, or those of the publisher, the editors and the reviewers. Any product that may be evaluated in this article, or claim that may be made by its manufacturer, is not guaranteed or endorsed by the publisher.
