# Supplementary material for: Study Design and Protocol of a Randomized Controlled Trial of the Efficacy of a Smartphone-Based Therapy of Migraine (SMARTGEM)
Source: Front Neurol. 2022 Jun 16;13:912288. doi: 10.3389/fneur.2022.912288 (PMC9243352; doi:10.3389/fneur.2022.912288)
Supplement: Supplementary file 1 [file Table_1.docx]

Supplementary Material

**Supplementary Table A.1 - Inclusion and exclusion criteria**

| *Inclusion criteria* | 1. Capability and willingness to give written informed consent. |
| --- | --- |
|  | 1. The participant is insured with a German statutory health insurance. |
|  | 1. Diagnosed with episodic migraine and at least 5 days of migraine per month or diagnosed with a chronic migraine with or without aura. Participants are diagnosed with migraine according to the ICHD-3 (The international classification of headache disorders, 3^rd^ edition) criteria (33). |
|  | 1. Beginning of migraines before the age of 50 and occurrence for at least one year. |
|  | 1. Less than three prior visits to the headache centres of the Charité – Universitätsmedizin Berlin (Charité), University Clinic Rostock or University Clinic Halle. |
|  | 1. Ownership of a smartphone compatible with Android or iOS, access to mobile internet and a basic understanding of apps. |
|  | 1. Adherence of at least 80% for the headache diary function of M-sense during 28 days prior to the first consultation in the headache centre. |
| *Exclusion criteria* | 1. Use of the commercially available version of the app (‘M-sense active’) for more than 14 days. |
|  | 1. Headache diseases other than migraine or tension type headache (≤ 50% of overall headache days). |
|  | 1. Headache diseases other than migraine or tension type headache (≤ 50% of overall headache days). |
|  | 1. Prior or current drug or alcohol abuse within one year prior to visit (excessive or compulsory use as determined by the study physician). |
|  | 1. Current use of medications, which, in the opinion of the study physician, indicate signs of abuse or dependence (exception analgesic or triptan overuse). |
|  | 1. Consumption of more than 3 doses/month of opioids or barbiturates. |
