# Supplementary material for: Study Design and Protocol of a Randomized Controlled Trial of the Efficacy of a Smartphone-Based Therapy of Migraine (SMARTGEM)
Source: Front Neurol. 2022 Jun 16;13:912288. doi: 10.3389/fneur.2022.912288 (PMC9243352; doi:10.3389/fneur.2022.912288)
Supplement: Supplementary file 2 [file Table_2.docx]

Supplementary Material

**Supplementary Table A.2 - Exploratory endpoints**

|  | Baseline vs 3 months | Baseline vs 6 months | Baseline vs 9 months | Baseline vs 12 months |
| --- | --- | --- | --- | --- |
| Migraine days per 28 days | x |  | x | x |
| Responders (patients with ≥ 30% reduction in headache frequency) | x | x | x | x |
| Headache days per 28 days | x |  | x | x |
| Days of acute medication intake | x |  | x | x |
| NRS (1-10) | x | x | x | x |
| Days with NRS > 5 | x | x | x | x |
| DASS | x | x | x | x |
| HIT-6 | x |  | x | x |
| EQ-5D-5L | x |  | x | x |
| PROMIS 29 | x |  | x | x |
| MARS-D | x | x | x | x |

DASS - Depression Anxiety Stress Scale; HIT-6 - Headache Impact-Test 6; PROMIS 29 - Patient-Reported Outcome-measurement Information System; NRS - Numerical Rating Scale; MARS-D - Medication Adherence Report Scale
